# Medication use and obesity in Brazil: results from the National Health Survey

**DOI:** 10.1038/s41598-020-76058-6

**Published:** 2020-11-02

**Authors:** Karina Abibi Rimes-Dias, Daniela Silva Canella

**Affiliations:** 1 Postgraduate Program in Food, Nutrition and Health, Rio de Janeiro State University, Rio de Janeiro, RJ Brazil; 2 Institute of Nutrition, Rio de Janeiro State University, Rio de Janeiro, RJ Brazil

**Keywords:** Risk factors, Nutrition disorders, Epidemiology

## Abstract

Noncommunicable diseases (NCDs) associated with obesity generally require drug treatment. The use of medications in individuals with obesity has not been extensively investigated. The objective of this study was to analyze the relation between obesity and medication use. Data from the Brazilian National Health Survey 2013 was used, including 59,402 individuals. Weight and height measures were used to calculate body mass index (BMI) and categorized individuals according to BMI classification (exposure). The number of medications used for treating nine obesity-related NCDs was the outcome variable. Multinomial regression analyses were performed. The risk of use of medications to treat at least one NCD increased progressively with rising BMI, where this risk was even higher for treating two or more diseases. The risk of having to treat two or more NCDs with medications was approximately 70% greater among individuals with overweight (adjusted RR = 1.66; 95%CI 1.46–1.89), 170% greater in those with class I obesity (adjusted RR = 2.68; 95%CI 2.29–3.12), 340% greater for class II obesity (adjusted RR = 4.44; 95%CI 3.54–5.56) and 450% greater among individuals with class III obesity (adjusted RR = 5.53; 95%CI 3.81–8.02), compared with normal-weight subjects. Obesity was directly associated with drug utilization and the number of medications used to treat obesity-related NCDs.

## Introduction

In the context of noncommunicable diseases (NCDs), obesity stands out for being both a disease and a risk factor for other NCDs, such as hypertension and diabetes. Thus, of the main NCDs affecting the world population, most are associated with obesity^1,2^. Currently, NCDs are the leading cause of death and disability in Brazil and globally, significantly reducing the quality of life of individuals with these conditions. These diseases are emerging at an increasingly younger age, impacting the more productive life years^3^.

The prevalence of obesity is rising steadily in practically all countries in the world, having tripled in the last four decades^3^. This increase in obesity has been accompanied by a rise in prevalence of its consequences, such as morbidity due to NCDs^1,2^ and economic burden, considering the expenses for health systems and for families^4–8^. The prevention, treatment and control of these diseases, together with their risk factors, depends on a set of health actions, including the use of medications^1^, one of the most common therapeutic interventions in patients with chronic diseases^1,9^.

Studies on the use of medications seek to elucidate the characteristics of users and also identify the factors associated with their use, helping to qualify this use and rationalize health resources^9,10^. Knowledge on the consequences of obesity can help effective strategies for disease management to be devised^11^.

Studies on the association between obesity and use of medications for NCDs associated with the disease are scarce worldwide, mainly in low- and middle-income countries, being limited to small studies. The objective of the present study was to analyze the association between obesity and medications use, based on representative data for the Brazilian population.

## Methods

### Study design and data source

A cross-sectional study was performed of secondary data from the Brazilian National Health Survey (*Pesquisa Nacional de Saúde*—PNS)—a population-based household survey conducted in Brazil in 2013 by the Brazilian Institute of Geography and Statistics (IBGE) in partnership with the Ministry of Health. The main objective of PNS was to produce nation-level data about the health status and lifestyles of the Brazilian population^12^.

### Study population and sampling

The National Health Survey assessed a representative sample of the Brazilian population aged 18 years old or more, comprising dwellers of private households situated nationwide^12^. Three-stage cluster sampling was employed. The first stage involved census tracts (Primary Sampling Units—PSU); the second, households; and the third, dwellers aged 18 years old or more residing in randomly selected households.

A minimum sample size of 1800 households per PSU was established for an estimated sample of 81,167 households. Losses were defined as: household closed or empty, refusal of dweller to engage with the interviewer and failure to conduct interview after three or more attempts, despite scheduling the visits. Upon conclusion of the field work, a total of 69,994 households were occupied, where 64,348 household interviews and 60,202 individual interviews were carried out. The complete information regarding the sampling is available in previous publications^12–14^.

### Data collection

Data collection for the PNS was carried out by the IBGE. The questionnaire used was subdivided into three parts: (1) household; (2) about all household dwellers; and (3) individual. The questionnaires for the household and for all dwellers were completed by only one resident who was able to report the socioeconomic and health status of the other residents of the household. The individual questionnaire was answered by only one dweller, aged at least 18 years, randomly selected using a hand-held personal computer (PDA—Personal Digital Assistant). The information collected was stored on the PDA. A detailed description of the collection process is available from the IBGE (2014)^12^.

Height and weight measurements were taken using a set of digital scales and portable stadiometer. Final measured weight and height were based on the arithmetic mean of the first and second measures taken^12^. The anthropometric measurements were taken adopting the same procedures as those used by the 2008–2009 Household Budget Survey^15^, also conducted by the IBGE in partnership with the Ministry of Health. All collection agents were trained to carry out the interviews using the PDA and were duly qualified to take all of the measurements^12^.

### Study variables

The main study variables were use of medications to treat NCDs related to obesity and body mass index (BMI) of the individuals.

The following groups of chronic diseases were surveyed about their diagnosis and treatment: systemic arterial hypertension, cardiovascular disease, stroke, diabetes mellitus, arthritis and rheumatism, chronic kidney disease, lung disease, chronic back problem, and depression^2,16–19^. For the treatment of hypertension and diabetes, information on the use of medications in the two weeks leading up to the study was collected, whereas, for the other diseases, the use of medications was investigated as the current way of treatment^12^. Individuals that referred to the diagnosis of these NCDs were asked about the kind of treatment adopted and use of medication was one of the possible answers.

The outcome variable of the study was “number of NCDs related to obesity treated with medications”, considering the sum of the answers “yes” for medication treatment of the nine groups of NCDs investigated. It was used as a proxy for the number of medications used since the questions do not include the number of medications used to treat each NCD. The variable was categorized into: no NCD treated with medication, one NCD treated with medication, and two or more NCDs treated with medication.

BMI classification of the individuals was the exposure variable of main interest in the present study. Based on weight and height measurements taken, BMI (kg/m^2^) was calculated and classified into: underweight (BMI < 18.5), normal weight (BMI 18.5–24.9), overweight (BMI 25.0–29.9), obesity class I (BMI 30.0–34.9), obesity class II (BMI 35.0–39.9) and obesity class III (BMI ≥ 40.0), as defined by the World Health Organization^20^. BMI was also analyzed in continuous basis. All individuals that had completed the individual section of the PNS questionnaire and provided weight and height data were eligible. Women who stated they were pregnant were excluded from the analyses.

The following sociodemographic and geographic variables were assessed: region (North, Northeast, Southeast, South, Mid-West), area (urban/rural), sex (male/female), age (stratified into: 18–29/30–39/40–49/50–59/ ≥ 60 years), race/skin color (white, black, yellow, mixed-race, indigenous) and education (stratified into: no formal education or primary incomplete, primary complete or secondary incomplete, secondary complete or higher incomplete, higher complete or greater).

### Data analysis

Descriptive statistical analysis of all variables was performed to determine the percentage distribution of the characteristics of interest in the study population. Relative frequencies were calculated to analyze the BMI classification and percentage of individuals reporting use of medications to treat each of the nine NCDs investigated, according to sociodemographic and geographic characteristics. In addition, the relative frequency of individuals who reported using medications for NCDs associated with obesity was described according to BMI classification, as well as the relative frequency of number of NCDs related to obesity treated with medications, according to BMI classification and age (considering the population as a whole, and stratified by adults and elderly). Differences were deemed statistically significant when there was no overlapping of confidence intervals (95%CI).

Associations between the number of obesity related NCDs treated with medications (outcome) and BMI classification (and also BMI continuous) (exposure) was determined using crude and adjusted multinomial regression (by region, area, sex, age and education), with calculations of relative risks (RR) (crude and adjusted) and their respective 95% confidence intervals (95%CI).

All data analyses were carried out using the statistics package Stata version 12.1 (Stata Corp., College Station, USA), with the survey module which takes into account the effects of complex sampling of the PNS.

### Ethical aspects

We declare that PNS and our analyses were carried out in accordance with the relevant national and international guidelines and regulations. The Brazilian National Health Survey was approved by the National Commission on Research Ethics for Humans (Comissão Nacional de Ética em Pesquisa—CONEP) of the National Board of Health, which regulates and approves health research involving human participants (CONEP protocol nº 328,159, June 26th, 2013). All study participants signed the Free and Informed Consent Form. We used data from the PNS, collected by the IBGE, and available online (https://www.ibge.gov.br/estatisticas/sociais/saude/9160-pesquisa-nacional-de-saude.html?=&t=microdados). The information contained in the database is confidential since specific data about each household such as identification of the household members, address and telephone are excluded.

Additionally, this study was reported in accordance with the Strengthening the Reporting of Observational Studies in Epidemiology (STROBE) checklist.

## Results

The population studied consists of 60,202 individuals aged 18 years or older. Of the original sample, 800 individuals (1.3%) were excluded for missing weight and/or height data, giving a total sample size of 59,402. Weight ranged from 30.0 kg to 179.0 kg, mean 70.4 kg (± 15.2 kg) while height ranged from 1.25 m to 2.03 m, mean 1.63 m (± 9.7 m). The final sample comprised individuals aged 18–109 years with BMI ranging from 13.1 to 64.6 kg/m^2^ (mean 26.5 kg/m^2^).

The majority of the population (57.2%) had excess weight, of which around a fifth were people with obesity (20.8%). Higher obesity rates were found among individuals from the Southern part of the country, who resided in rural areas, were female, middle-aged (rising prevalence from age 30 years and older), black skin color/race and those with lower education (no formal education or incomplete primary level) (Table 1).

**Table 1:** Distribution (%) of sociodemographic and geographic characteristics of study population, by BMI classification (n = 59,402). National Health Survey, Brazil, 2013.

Sociodemographic and Geographic Characteristics	BMI classification^a^ (%)					
	Underweight	Normal weight	Overweight	Obesity I	Obesity II	Obesity III
**Brazil**	2.5	40.3	36.4	14.9	4.3	1.6
**Region**						
North	2.8	43.6	36.2	13.2	3.1	1.2
Northeast	3.1	43.6	35.4	13.3	3.3	1.3
Southeast	2.2	39.4	36.3	15.5	4.7	1.8
South	2.0	37.8	36.9	16.2	5.2	1.8
Mid-west	2.6	40.5	35.0	15.4	4.5	2.0
**Area**						
Urban	3.0	43.9	36.2	13.0	2.9	0.9
Rural	2.5	42.8	35.1	14.7	3.6	1.3
**Sex**						
Male	2.1	42.3	38.8	12.8	3.1	0.8
Female	2.8	39.2	33.7	16.6	5.3	2.4
**Age (years)**						
18–29	4.7	56.5	26.4	8.6	2.7	1.0
30–39	1.8	39.9	37.8	13.8	4.8	2.0
40–49	1.2	33.3	40.7	18.0	4.8	1.9
50–59	1.3	30.5	40.9	19.8	5.4	2.1
≥ 60	2.6	35.1	39.0	17.3	4.5	1.6
**Race/skin color**						
White	2.3	38.7	36.7	15.7	4.7	1.8
Black	2.3	41.5	32.9	16.5	4.7	2.0
Yellow	1.4	53.9	33.0	7.4	2.9	1.4
Mixed-color	2.8	42.4	36.1	13.6	3.8	1.4
Indigenous	2.0	41.7	36.0	13.8	4.1	2.3
**Educational level**						
No formal education or primary incomplete	2.5	38.7	36.7	15.8	4.5	1.8
Primary complete or secondary incomplete	2.8	43.6	33.6	13.7	4.4	1.8
Secondary complete or higher incomplete	2.8	41.9	35.5	14.0	4.0	1.8
Complete higher education or greater	1.4	39.9	38.3	15.2	4.2	0.8

^a^Underweight: BMI < 18.5 kg/m^2^. Normal weight: BMI 18.5–24.9 kg/m^2^. Overweight: BMI 25.0–29.9 kg/m^2^. Obesity I: BMI 30.0–34.5 kg/m^2^. Obesity II: BMI 35.0–39.9 kg/m^2^. Obesity III: BMI ≥ 40 kg/m^2^.

Regarding the nine groups of NCDs associated with obesity analyzed in the present study, a greater percentage of medication use was reported for the treatment of: arterial hypertension (17.4%), chronic back problems (7.4%), diabetes mellitus (4.7%) and depression (4.0%). Despite the variations observed in the frequencies, no differences in these rates was found for the sociodemographic and geographic variables analyzed (Table 2).

**Table 2:** Relative frequency of individuals reporting use of medications for noncommunicable diseases (NCDs) related to obesity, by disease group and sociodemographic and geographic characteristics (n = 60,202).

Sociodemographic and geographic characteristics	Individuals reporting use of medications, by group of NCDs related to obesity (%)								
	Hypertension	DM	CVD	Stroke	CKD	Lung disease	Arthritis or rheumatism	Chronic back pain	Depression
**Brazil**	17.4	4.7	2.6	0.8	0.8	0.8	3.2	7.4	4.0
**Region**									
North	10.4	3.1	1.2	1.0	0.8	0.4	2.7	6.8	1.3
Northeast	15.3	3.9	1.8	0.8	0.8	0.2	3.0	8.0	2.4
Southeast	19.4	5.5	2.9	0.8	0.7	1.0	3.0	6.2	4.6
South	19.1	4.4	3.7	0.9	1.2	1.5	4.2	9.9	6.5
Mid-west	17.0	4.6	2.8	0.7	0.7	0.7	3.4	7.8	3.3
**Area**									
Urban	12.3	1.5	1.5	1.0	0.6	0.3	2.9	8.3	1.6
Rural	14.2	2.0	2.0	1.1	0.8	0.3	2.4	6.3	2.2
**Sex**									
Male	14.2	4.0	2.6	0.9	0.7	0.8	1.6	5.7	2.0
Female	20.4	5.3	2.6	0.7	0.9	0.8	4.6	8.9	5.7
**Age (years)**									
18–29	1.0	0.2	0.1	0.02	0.3	0.6	5.3	2.0	1.7
30–39	6.1	0.9	0.6	0.1	0.3	0.3	9.4	5.5	3.5
40–49	15.5	3.4	1.4	0.5	0.9	0.7	2.4	9.6	5.3
50–59	29.2	7.8	4.1	1.1	1.2	0.6	5.9	11.3	5.5
**Race/skin color**									
White	18.7	5.2	3.1	0.9	0.8	1.1	3.5	7.5	4.7
Black	19.3	5.5	2.0	0.8	0.8	0.4	3.3	7.5	2.8
Yellow	15.6	6.0	2.6	0.02	0.4	0.6	1.8	4.6	2.5
Mixed-race	15.6	3.9	2.1	0.7	0.8	0.6	2.8	7.2	3.5
Indigenous	14.1	5.0	3.8	0.1	1.6	0.9	2.9	15.7	5.6
**Educational level**									
No formal education or primary incomplete	26.1	7.0	4.4	1.5	1.3	1.0	4.7	11.0	4.6
Primary complete or secondary incomplete	13.3	4.2	2.1	0.5	0.6	0.7	3.0	6.1	3.6
Secondary complete or higher incomplete	10.1	2.6	1.0	0.4	0.5	0.5	1.8	4.8	3.2
Complete higher education or greater	14.8	3.5	1.7	0.3	0.5	0.9	2.3	4.6	4.3

National Health Survey, Brazil, 2013.

DM, diabetes mellitus; CVD, cardiovascular disease; CKD, chronic kidney disease.

The number of diseases treated with medications reported on an individual level ranged from zero to seven different diseases (data not shown). Individuals with greater BMI used more medications for treating their NCDs, namely: hypertension, diabetes, cardiovascular disease, arthritis and rheumatism, chronic back problems, and depression, where this number increased progressively with rising BMI for all diseases cited (Table 3).

**Table 3:** Relative frequency of individuals reporting use of medications for noncommunicable diseases (NCDs) related to obesity, by BMI classification (n = 59,402).

BMI classification^a^	Hypertension		DM		CVD		Stroke		CKD		Lung disease		Arthritis or rheumatism		Chronic back problem		Depression	
	Individuals reporting use of medications, by group of NCDs related to obesity																	
	%	95%CI	%	95%CI	%	95%CI	%	95%CI	%	95%CI	%	95%CI	%	95%CI	%	95%CI	%	95%CI
Underweight	6.9	4.8–8.9	2.6	2.3–2.9	2.0	1.7–2.3	0.6	0.5–0.8	0.7	0.5–0.9	0.6	0.5–0.8	2.2	1.8–2.5	5.8	5.3–6.4	3.0	2.6–3.4
Normal weight	10.2	9.6–10.9	1.5	0.7–2.2	1.6	0.4–3.7	0.2	0.0–0.4	0.5	0.0–0.9	1.5	0.5–2.5	1.4	0.5–2.3	4.8	3.0–6.5	3.1	1.6–4.7
Overweight	18.6	17.7–19.4	5.2	4.6–5.7	2.8	2.4–3.2	0.8	0.6–1.0	0.9	0.7–1.1	0.7	0.5–0.9	3.9	3.0–3.8	8.2	7.6–8.9	4.1	3.6–4.7
Obesity I	28.4	26.7–30.1	7.8	6.8–8.8	3.2	2.7–3.8	1.4	0.9–1.9	1.1	0.7–1.4	1.1	0.6–1.6	4.7	4.0–5.4	8.8	7.8–9.7	5.5	4.6–6.4
Obesity II	35.1	32.0–38.2	9.9	8.0–11.8	4.5	3.2–5.9	0.8	0.4–1.2	0.5	0.0–1.0	1.2	0.4–2.0	5.6	4.2–7.1	10.8	8.7–12.9	6.5	4.7–8.4
Obesity III	41.8	36.2–47.5	9.1	6.5–11.7	2.9	1.4–4.4	0.9	0.1–1.9	1.2	0.4–1.9	1.3	0.4–2.3	7.6	4.6–10.6	10.4	7.3–13.6	6.9	4.4–9.5

National Health Survey, Brazil, 2013.

^a^Underweight: BMI < 18.5 kg/m^2^. Normal weight: BMI 18.5–24.9 kg/m^2^. Overweight: BMI 25.0–29.9 kg/m^2^. Obesity I: BMI 30.0–34.5 kg/m^2^. Obesity II: BMI 35.0–39.9 kg/m^2^. Obesity III: BMI ≥ 40 kg/m^2^.

The frequency of individuals who reported using medications to treat one or more diseases also increased progressively with rising BMI, even after stratifying by age (adults and elderly). Approximately a fifth of individuals with class III obesity (20.2%) reported treating two or more diseases with medications, compared with only 5.6% of normal-weight individuals (14.6% difference). For the age groups (adults and elderly), the percentage difference between adults with class III obesity and normal-weight individuals reporting treating two or more diseases with medications was 13.9%, increasing to 17.1% among the elderly group (Table 4).

**Table 4:** Relative frequency of the number of noncommunicable diseases (NCDs) related to obesity treated with medications, by BMI classification and age (n = 59,402).

NCDs related to obesity treated with medications	BMI classification^a^											
	Underweight		Normal weight		Overweight		Obesity I		Obesity II		Obesity III	
	%	95%CI	%	95%CI	%	95%CI	%	95%CI	%	95%CI	%	95%CI
**Brazil**												
None	83.9	80.9–86.9	80.0	79.2–80.9	68.8	67.8–69.8	59.9	58.1–61.7	54.4	51.0–57.7	49.0	43.5–54.5
1	11.9	9.2–14.6	14.4	13.6–15.1	21.2	20.2–22.1	24.8	23.2–26.3	26.0	23.1–29.0	30.7	25.3–36.2
≥ 2	4.2	2.8–5.6	5.6	5.1–6.0	10.0	9.3–10.6	15.2	13.9–16.6	19.6	16.7–22.4	20.2	16.1–24.4
**Adults (18–59 years old)**												
None	89.2	86.1–92.2	86.2	85.4–87.0	76.6	75.6–77.6	68.2	66.2–70.2	60.3	56.6–63.9	54.2	48.1–60.3
1	8.6	5.8–11.3	11.2	10.4–12.0	17.6	16.7–18.6	22.4	20.7–24.2	24.2	21.0–27.5	29.3	23.2–35.4
≥ 2	2.6	1.0–3.4	2.6	2.3–3.0	5.7	5.2–6.3	9.4	8.2–10.6	15.5	12.8–18.2	16.5	12.5–20.4
**Elderly people (≥ 60 years old)**												
None	60.8	51.9–69.8	46.9	44.1–49.7	36.9	34.5–39.2	29.1	25.7–32.5	29.0	22.0–36.0	23.7	9.7–37.8
1	26.4	18.4–34.4	31.6	29.2–34.0	35.8	33.2–38.4	33.7	30.2–37.2	33.7	26.8–40.5	37.6	24.0–51.3
≥ 2	12.7	7.2–18.2	21.5	19.1–23.9	27.3	25.0–30.0	37.2	33.1–41.2	37.3	30.2–44.4	38.6	25.3–52.0

National Health Survey, Brazil, 2013.

^a^Underweight: BMI < 18.5 kg/m^2.^ Normal weight: BMI 18.5–24.9 kg/m^2^. Overweight: BMI 25.0–29.9 kg/m^2^. Obesity I: BMI 30.0–34.5 kg/m^2^. Obesity II: BMI 35.0–39.9 kg/m^2^. Obesity III: BMI ≥ 40 kg/m^2^.

The multinomial logistic regression results (crude and adjusted) showed that BMI classification was associated with the number of obesity-related comorbidities treated using medications across all categories, except (on crude model) underweight individuals. Taking “normal-weight individual” as the reference category, the risk of use of medications to treat at least one NCD rose progressively with increasing BMI, where this risk was even higher for treating two or more diseases, particularly after adjustment. The risk of treating two or more diseases with medications was approximately: 70% greater among individuals with overweight (adjusted RR = 1.66; 1.46–1.89), 170% greater among those with class I obesity (adjusted RR = 2.68; 2.29–3.12), 340% greater for class II obesity (adjusted RR = 4.44; 3.54–5.56) and 450% greater among individuals with class III obesity (adjusted RR = 5.53; 3.81–8.02), compared with normal-weight individuals. Individuals with severe obesity (class III) had an almost six-fold higher risk of having to took two or more medications to treat NCDs related to obesity than those of normal weight (Table 5). Complementary analysis using BMI on a continuous basis confirmed the statistical significance of the results (data not shown).

**Table 5:** Results of multinomial logistic regression for the association between the number of noncommunicable diseases (NCDs) related to obesity treated with medications and BMI classification (n = 59,402).

BMI classification^a^	Number of NCDs related to obesity treated with medications							
	Crude relative risk				Adjusted relative risk^b^			
	1	95%CI	≥ 2	95%CI	1	95%CI	≥ 2	95%CI
Normal weight	Reference		Reference		Reference		Reference	
Underweight	0.79	0.60–1.04	0.72	0.50–1.05	0.78	0.58–1.06	0.63	0.41–0.96
Overweight	1.71	1.57–1.86	2.08	1.83–2.35	1.41	1.29–1.55	1.66	1.46–1.89
Obesity I	2.30	2.06–2.57	3.65	3.16–4.22	1.77	1.57–2.00	2.68	2.29–3.12
Obesity II	2.66	2.24–3.16	5.19	4.21–6.40	2.26	1.88–2.72	4.44	3.54–5.56
Obesity III	3.48	2.64–4.59	5.94	4.44–7.95	3.03	2.21–4.15	5.53	3.81–8.02

National Health Survey, Brazil, 2013.

^a^Underweight: BMI < 18.5 kg/m^2^. Normal weight: BMI 18.5–24.9 kg/m^2^. Overweight: BMI 25.0–29.9 kg/m^2^. Obesity I: BMI 30.0–34.5 kg/m^2^. Obesity II: BMI 35.0–39.9 kg/m^2.^ Obesity III: BMI ≥ 40 kg/m^2^.

^b^Model adjusted by region, area, sex, age and education.

## Discussion

Using data from a representative sample of the Brazilian population aged 18 years or older, an independent direct association between obesity and use of medications to treat NCDs was found. In addition, increasing BMI was correlated with a higher proportion of individuals using medications to treat hypertension, diabetes, cardiovascular disease, arthritis and rheumatism, chronic back problems, and depression. In 2013, over a fifth of the Brazilian population had obesity, representing a 6% increase in the rate of the disease in less than five years^15^. A rise in levels of obesity leads to a greater prevalence of related chronic comorbidies^1,18,19^ and greater necessity for use of medications to treat these diseases^9,21^. Curbing the rise in obesity is therefore an urgent public health priority in Brazil and worldwide^22,23^.

Data from the National Survey on Access, Use and Promotion of Rational Use of Medications in Brazil, conducted in 2013–2014, revealed that the majority of the Brazilian population (50.7%) used at least one medication, where in almost half of cases this was for treatment of NCDs^24^. Based on the link between medications use and presence of chronic disease, the findings of the present study, besides confirming the association between obesity and the development or worsening of different NCDs, showed the strength of this association, given that rise in BMI was associated, progressively, with the necessity for drugs treatment of these conditions. The PNS did not collect data on all groups of chronic diseases related to obesity reported in the literature (e.g. dyslipidemia and cancers), and hence this association may have been underestimated.

In the present study, the higher the BMI, the greater the risk of having to treat more than one NCD related to obesity with medications. Similar results were verified in Germany: individuals with severe obesity (class III) had an over four-fold higher probability of using medications to treat chronic diseases compared to those of normal weight^25^. The deleterious effects of overuse of medications have been extensively described in the literature, where chronic use increases the risk of iatrogenic events and functional decline in the organism^26^.

In addition to the health repercussions, greater use of medications by individuals with obesity may also have a financial impact both on society, owing to increased health care system costs, and on the individual and their family, as a result of out-of-pocket expediture^4,10^. The PNS did not survey the monetary cost of drug expenditures, but a previous national study also based on representative data for the Brazilian population, showed that the presence and increase in number of individuals with obesity residing at the household resulted in an increase in health spending, particularly expenditure on medications. The treatment of NCDs calls for long-term use of medications, making this a “fixed” expense in the family budget^21^. According to the World Health Organization, the importance of a national health system lies not only in improving the quality of life of its population, but also to protect them against the financial burden of diseases^20^. Effective obesity prevention programs for the whole population, and intervention programs for individuals with the disease, can help reduce the use of medications (and their costs) over the long term.

The results of our study also showed a reduction in the frequency of individuals with severe obesity (class III) who reported using medication among some of the groups of NCDs, which may indicate cases of early mortality in these groups, since the literature also points to association of obesity with the increased risk of mortality, and individuals diagnosed with NCDs would be even more vulnerable to this outcome. The small number of individuals in this category (1.6%) can also justify this lower frequency.

In the current scenario of a rapidly aging population and high prevalence of NCDs, elderly constitute the population strata with the highest use of medications. Considering medications used for eight chronic diseases surveyed by the National Survey on Access, Use and Promotion of Rational Use of Medications in Brazil, it was found that 18% of elderly used at least five drugs^21^. Intensifying this context, the present study results showed that, after stratifying for age (adults and elderly), elderly people with obesity used more medications to treat NCDs than their normal-weight counterparts. These findings corroborate the results of a U.S study involving data from over 57,500 individuals, in which male elderly with obesity, for instance, used 0.9 medications more than normal-weight individuals in 1988 and this difference had increased to 2.5 medications by 2012^27^. The findings of the present study also suggest that the rise in obesity can also promote an increase in the use of medications in this population.

Some NCDs may be more prevalent in underweight individuals than in those normal-weight. However, in this study, results related to underweight might be due to a relatively small number of cases (2.5%), compared with the other BMI categories. To reduce this uncertainty, sensitivity analysis was conducted grouping underweight and normal weight individuals and the association between BMI and medication use remains significant.

Despite the limitations inherent to a cross-sectional design study in terms of causal inference, an important strength is that the PNS involved a complex sampling process of data on over 60,000 non-institutionalized respondents. These factors allowed robust nationally representative estimates to be made for Brazil’s five regions, 27 Federal Units, and households located in both urban and rural areas. Given the high cost and operational challenges of collecting data for large and representative samples, using data from national surveys like the PNS represents an attractive alternative. It is also noteworthy that the PNS provides anthropometric measurements. The present investigation reports novel and consistent results on this issue from a middle-income country.

In the individual interviews, the timeframe for recalling medications use was relatively short (over the last 15 days or current treatment regimen). Nevertheless, the risk of forgetting or confounding the number used, particularly in the elderly population, should be recognized, since number of medications used and age are relevant factors when investigating memory bias. However, a previous study showed that chronic conditions, requiring continuous use of medications, are more likely to be reported in surveys than sporadic events (acute or seasonal diseases, accidents, and emergency surgery), thereby attenuating this type of bias^21^.

In the present investigation, it was not possible to assess more than one type of medication for treating the same disease. Consequently, the total number of medications used may have been underestimated, because in some cases it is necessary to use more than one type of medication to treat the same disease. This is the case for hypertension, for which at least two types of different drugs are often used in treatment (an anti-hypertensive and a diuretic agent). Another point to bear in mind is that the increase in medications use in Brazil might be attributed to improvements in policy enabling access to drugs through the National Health System (*Sistema Único de Saúde*—SUS), at least up until 2015^28,29^.

Lastly, despite the adjustment in the regression models, we recognize that the possibility of residual confounding can not be discarded. Education was used as an income proxy and it was not possible to assess medication access, which may interfere in the use.

The treatment and control of NCDs related to obesity calls for protocols that incorporate findings on greater use of medications (to treat these diseases) by individuals with obesity. This approach can help promote actions that address issues ranging from drug access to rationalizing medication use in this specific population, readily identifiable in the health system. The management of obesity and the clinical practice (medical and multiprofessional) should also be influenced by this knowledge, seeking ways of managing these associated chronic morbidity and improving its control, preventing adverse effects related with the chronic use of medications, like unnecessary hospital admissions and ER visits, and above all, progressive disability of the individual for everyday activities, with reduction or loss of independence and autonomy. For individuals who already have obesity, specific actions can attenuate the disease impact and prevent even greater medications use over time. As this greater use translates to harmful effects and high costs on health, it should be incorporated as priority in the national health system planning agenda, both in the public and private spheres.

The findings of the present study revealed obesity was associated with both the presence and number of NCDs treated with medications. Use of medications among individuals with obesity was high and rose progressively with increasing BMI. Individuals with obesity should therefore be monitored carefully with regard to use of medications. Further studies with different designs, such as longitudinal, and investigations exploring medications access and expenditures should be carried out.
